# Breath‐by‐breath measurement of alveolar gas exchange must preserve mass balance and conform to a physiological definition of a breath

**DOI:** 10.1113/EP092221

**Published:** 2025-02-21

**Authors:** Michele Girardi, Carlo Capelli, Carrie Ferguson, Susan A. Ward, Harry B. Rossiter

**Affiliations:** ^1^ Institute of Respiratory Medicine and Exercise Physiology, Division of Respiratory and Critical Care Physiology and Medicine The Lundquist Institute for Biomedical Innovation at Harbor‐UCLA Medical Center Torrance California USA; ^2^ Department of Pathophysiology and Transplants University of Milano Milano Italy; ^3^ Human Bio‐Energetics Research Centre Crickhowell Wales UK

**Keywords:** breathing cycle, CPET, gas exchange, kinetics

## Abstract

Tidal breathing in awake humans is variable. This variability causes changes in lung gas stores that affect gas exchange measurements. To overcome this, several algorithms provide solutions for breath‐by‐breath alveolar gas exchange measurement; however, there is no consensus on a physiologically robust method suitable for widespread application. A recent approach, the ‘independent‐breath’ (IND) algorithm, avoids the complexity of measuring breath‐by‐breath changes in lung volume by redefining what is meant by a ‘breath’. Specifically, it defines a single breathing cycle as the time between equal values of the $F_{O_{2}}$/$F_{N_{2}}$ (or $F_{CO_{2}}$/$F_{N_{2}}$) ratio, that is, the ratio of fractional concentrations of lung‐expired O_2_ (or CO_2_) and nitrogen (N_2_). These developments imply that the end of one breath is not, by necessity, aligned with the start of the next. Here we demonstrate how the use of the IND algorithm fails to conserve breath‐by‐breath mass balance of O_2_ and CO_2_ exchanged between the atmosphere and tissues (and vice versa). We propose a new term, within the IND algorithm, designed to overcome this limitation. We also present the far‐reaching implications of using algorithms based on alternative definitions of the breathing cycle, including challenges in measuring and interpreting the respiratory exchange ratio, pulmonary gas exchange efficiency, dead space fraction of the breath, control of breathing, and a broad spectrum of clinically relevant cardiopulmonary exercise testing variables. Therefore, we do not support the widespread adoption of currently available alternative definitions of the breathing cycle as a legitimate solution for breath‐by‐breath alveolar gas exchange measurement in research or clinical settings.

## Abstract (highlights)

- **What is the central question of this study?**
  The IND algorithm for breath‐by‐breath alveolar gas exchange computation redefines ‘a breath’: what are the implications of this approach for the measurement of gas exchange at rest and during exercise?
- **What is the main finding and its importance?**
  The IND algorithm does not maintain continuity between consecutive breaths, violating the conservation of mass for breath‐by‐breath alveolar gas exchange measurements. A volume correction to the algorithm is provided to address this discrepancy and preserve mass balance. However, by redefining the conventional breathing cycle, the IND algorithm has additional implications for cardiopulmonary exercise testing interpretation because variables with demonstrated diagnostic and prognostic value cannot be accurately determined.

## 1 MEASUREMENT OF BREATH‐BY‐BREATH ALVEOLAR GAS EXCHANGE

The advent of computerized breath‐by‐breath (B‐by‐B) whole‐body gas exchange measurement (Beaver et al., 1973; Ward, 2018) has advanced knowledge of pulmonary gas exchange and control of breathing responses during exercise and propelled the clinical diagnostic and prognostic utility of cardio‐pulmonary exercise testing (CPET). The adoption of alveolar, as opposed to mouth or whole‐body, gas exchange measurements has the potential to extend these advances by focusing on the actual site of alveolar–capillary gas exchange. However, while the optimal methods for B‐by‐B calculation of alveolar gas exchange have been debated for over 40 years (reviewed in Capelli et al., 2011; Girardi et al., 2023; Ward, 2018; Whipp et al., 2005), a solution suitable for widespread application has been elusive.

The total volume of the alveolar–capillary O_2_ transferred over a breath *i* ($V_{O_{2i}A}$) may be computed by subtracting the change in lung gas storage, occurring between the breath *i* and the preceding breath *i* − 1 (Δ $V_{O_{2i}S}$), from the O_2_ taken up at the mouth ($V_{O_{2i}M}$):

(1) $$V_{O_{2i}A}=V_{O_{2i}M}-\Delta V_{O_{2i}S}$$

A similar equation for the alveolar–capillary CO_2_ volume transferred over a breath *i* ($V_{CO_{2i}A}$) can be written by substituting CO_2_ for O_2_ in Equation 1 and adding the change in lung CO_2_ stores to the output at the mouth (Beaver et al., 1981). For simplicity, note that Equation 1 (and the subsequent equations) refers to volumes rather than rates of gas exchange. It is important to note that current commercial B‐by‐B systems do not measure instantaneous gas‐flow temperature and humidity throughout the breathing cycle (Macfarlane, 2001; Ward, 2018). The use of drying tubing (e.g., Nafion tubing) aims to mitigate the influence of water vapour pressure, so that it approaches that of the local atmosphere before gas concentrations are measured (Namieśnik & Wardencki, 1999). However, the effects of rapid fluctuations in gas temperature and humidity throughout the breathing cycle on gas flow are not measured (for details see Beaver, 1973; Macfarlane, 2001; Ward, 2018). Rather, the assumption is made that expired gas flow is fully saturated at body temperature, or near‐body temperature, and this assumption will contribute to the error in gas volume calculations (Beaver, 1973; Macfarlane, 2001; Ward, 2018).

**FIGURE 1:**
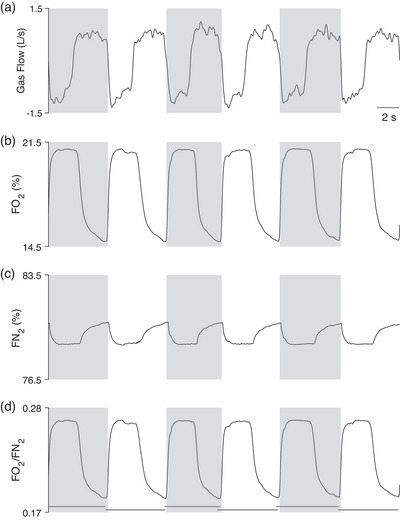
(a) Gas flow, where positive deflections occur during the expiration phase and negative deflections during the inspiration phase. The boundaries of the grey and white areas represent a single breathing cycle according to the classical definition (i.e., the time interval between the start of two consecutive inspiration phases). (b) Intra‐breath trace of oxygen fractional concentration ($F_{O_{2}}$). (c) Intra‐breath trace of nitrogen fractional concentration ($F_{N_{2}}$). Of note, $F_{N_{2}}$ is not typically measured directly by commercial breath‐by‐breath systems; rather, it is estimated as $F_{N_{2}}$ = 1 − ($F_{O_{2}}$ + $F_{CO_{2}}$). Accurate estimation of $F_{N_{2}}$ requires properly aligned intra‐breath traces of $F_{O_{2}}$ and $F_{CO_{2}}$, necessitating the use of fast‐response O_2_ and CO_2_ sensors. Misalignment introduces distortion in the estimated $F_{N_{2}}$ trace, especially during inspiration–expiration transitions (and vice versa). (d) Intra‐breath trace of the $F_{O_{2}}$ to $F_{N_{2}}$ ratio. This trace is used to identify the breathing cycle when applying the IND algorithm. The panel shows the breathing cycles identified by the IND algorithm (thick horizontal black line) and those identified by the classical definition (grey and white areas). Notably, breathing cycles identified using the IND algorithm do not coincide with those identified by standard methods, and there may be either an overlap or a gap between breaths (see Figure 2). Traces were collected and edited from a healthy, physically active female adult during steady‐state moderate‐intensity work‐rate cycle ergometry.

Solutions to determine the change in lung gas stores in Equation 1, and hence calculate alveolar gas exchange, are complex. Recent proposed solutions: (1) challenge the preservation of mass balance of gas flow between the mouth, the alveoli and pulmonary–capillary blood, and (2) change the definition of a ‘breath’, such that a single breathing cycle no longer constitutes the time between the onset of two consecutive inspirations (reviewed in Girardi et al., 2023; Figure 1). We propose that the consequent implications for quantification of key variables that rely on ventilatory‐based measurements lead to a logical impasse by introducing major interpretational complications that challenge future physiological advances.

The ‘independent‐breath’ (IND) algorithm is a relatively new approach for measuring B‐by‐B alveolar gas exchange (Cettolo & Francescato, 2018; Francescato & Cettolo, 2019, 2024a). Previous approaches required knowledge of end‐expiratory lung volume, on a B‐by‐B basis (reviewed in Capelli et al., 2011; Girardi et al., 2023). The IND algorithm was designed to circumvent this complexity by using specific criteria that are identified without considering continuity between consecutive inspiratory–expiratory phases (Cettolo & Francescato, 2018; Francescato & Cettolo, 2019). Hence, the volume of alveolar O_2_ uptake over a breath *i* can be calculated:

(2) $$V_{O_{2i}A}=\int_{t1_{i}}^{t2_{i}}\left({\dot{V}}_{I}-{\dot{V}}_{E}\right)\cdot F_{O_{2}}\cdot dt-\frac{F_{O_{2}\left(t1_{i}\right)}}{F_{N_{2}\left(t1_{i}\right)}}\cdot \int_{t1_{i}}^{t2_{i}}\left({\dot{V}}_{I}-{\dot{V}}_{E}\right)\cdot F_{N_{2}}\cdot dt$$

**FIGURE 2:**
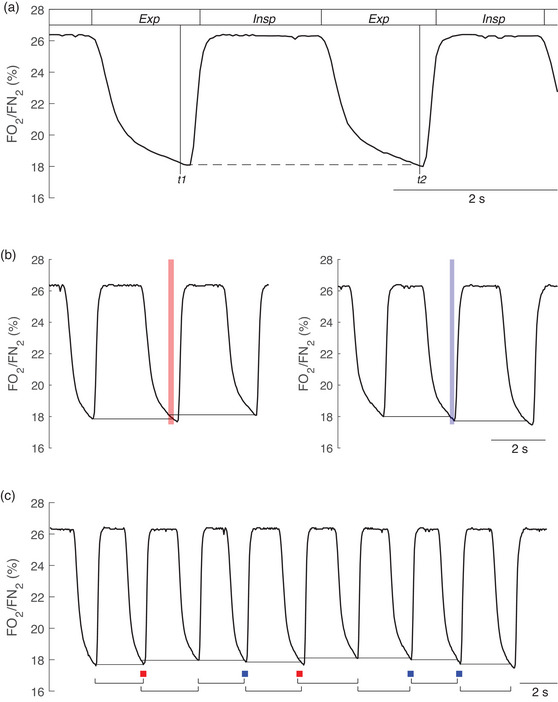
(a) For the IND algorithm, the reference value *t*_1_ is chosen according to specific criteria, whereas *t*_2_ is chosen to yield $F_{O_{2}}$/$F_{N_{2}}$ equal to that at *t*_1_. The breathing cycle is defined as the period elapsing between *t*_1_ and *t*_2_. ‘Exp’ and ‘Insp’ mark the expiration and inspiration phases, respectively, of a conventional breath. (b) Example of overlaps and gaps between breathing cycles identified using the IND algorithm. The red and blue vertical areas represent the overlap and gap between two breaths, respectively. (c) Series of breathing cycles with overlaps and gaps identified using the IND algorithm. The red and blue squares depict when an overlap or gap occurs, respectively. Traces were collected and edited from a healthy, physically active male adult during steady‐state moderate‐intensity work‐rate cycle ergometry.

In Equation 2 ${\dot{V}}_{I}$ and ${\dot{V}}_{E}$ represent the instantaneous absolute inspired and expired flow rates, while $F_{O_{2}}$ and $F_{N_{2}}$ are the instantaneous fractional gas concentrations of O_2_ and N_2_ (Figure 1). Likewise, simple modifications to Equation 2 allow measurement of alveolar CO_2_ output (Girardi et al., 2023). Using the IND algorithm, the start of the *i*th breath (at time *t*_1_) is chosen based on specific criteria, and the end of the *i*th breath (at time *t*_2_) is chosen to yield $F_{O_{2}}$/$F_{N_{2}}$ equal to that at *t*_1_ (i.e., $\frac{F_{O_{2}\left(t1_{i}\right)}}{F_{N_{2}\left(t1_{i}\right)}}=\frac{F_{O_{2}\left(t2_{i}\right)}}{F_{N_{2}\left(t2_{i}\right)}}$) (Cettolo & Francescato, 2018; Francescato & Cettolo, 2019). The time interval between *t*_1_ and *t*_2_ then represents the total time of a given breathing cycle. Thus, for the IND algorithm, this breathing cycle need not, and in fact commonly does not, coincide with the time between the beginning of two consecutive inspirations based on the conventional definition of the breathing cycle (Figures 1d and  2a).

With the IND algorithm, each ‘breath’ has its own *t*_1_ and *t*_2_, where the start of breath *i* does not necessarily align with the end of the preceding one, leading to non‐contiguity in the timing of breathing cycles (Figures 2b and 2c). Consequently, some breathing cycles may ‘overlap’ with each other, representing a small proportion (∼4%) of the volume of O_2_ taken up into the alveolar compartment that is counted twice (Cettolo & Francescato, 2018). In other cycles, there may be gaps between consecutive breaths, where a small volume of alveolar O_2_ uptake goes unmeasured (Figures 2b and 2c). At rest, these errors can be as much as approximately ±20% of the identified *i*th breath duration, rising to +30% during voluntary hyperventilation and falling to −50% immediately after hyperventilation (Cettolo & Francescato, 2018).

**FIGURE 3:**
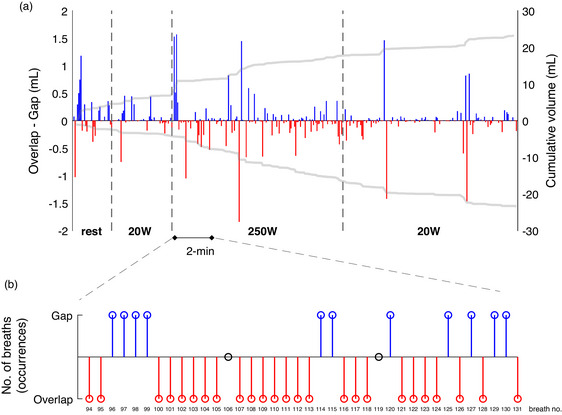
(a) O_2_ volume computed during each overlap (red) and gap (blue) observed during high‐intensity step‐wise exercise transition. The protocol consisted of 3 min of rest, followed by 6 min at 20 W, 6 min at 250 W (which was above critical power for this individual), and 6 min recovery at 20 W. Grey lines represent the cumulative O_2_ volume for overlaps (bottom) and gaps (top). The IND algorithm identified a total of 412 breathing cycles, of which 376 presented with gaps and overlaps. Note the uneven distribution of gaps and overlaps immediately at the onset and offset of the step transitions. Despite the same exercise duration (i.e., 6 min), the total number of overlaps and gaps differed between the pre‐ and post‐250 W bouts (pre‐bout: gaps *n* = 30, overlaps *n* = 21; 250 W bout: gaps *n* = 55, overlaps *n* = 91; post‐bout: gaps *n* = 68, overlaps *n* = 79). Nineteen gaps and 13 overlaps were identified during the initial 3 min of rest. (b) Zoomed‐in view of the first 2 min of the onset of the step‐wise transition phase (overlaps in red, gaps in blue, breaths without gaps/overlaps in black). Note the prevalence of overlaps during this phase.

An example of the effect of gaps and overlaps from a high‐intensity constant power cycling test (3 min rest, 6 min at 20 W, 6 min at 250 W, and 6 min recovery at 20 W) is shown in Figure 3. In this example, using the IND algorithm resulted in 376 out of 412 breathing cycles (91.3%) with gaps (172 cycles) or overlaps (204 cycles), and introduced errors in O_2_ volume exchanged ranging from 0% to 3% for gaps and 0% to 2% for overlaps. Consistent with previous findings (Cettolo & Francescato, 2018), the O₂ volume during gaps and overlaps is relatively small (Figure 3a). Nevertheless, their presence introduces error, and fails to preserve mass balance in gas exchange measurements.

The authors of the IND algorithm recognize these potential errors, but argue they are justified by the fact that the known error is typcially negligible and offset by the additional insight gained through reducing B‐by‐B variability and providing a proximal estimate of $V_{O_{2i}A}$. However, the extent of these potential errors is under‐researched, and it is currently unknown whether they may lead to physiologically meaningful errors in $V_{O_{2i}A}$ measurement during exercise, especially in non‐steady‐state exercise conditions when rapid changes in lung gas stores can occur (Capelli et al., 2011). As our example exercise test in Figure 3 demonstrates, errors for individual breaths can be substantial, and are unevenly distributed during transient phases. Figure 3b highlights a greater prevalence of overlaps than gaps during the first 2 min of the transition from 20 to 250 W. Similar findings were observed during the offset transition phase (Figure 3a). The greater prevalence of overlaps results from rapid changes in the composition of expired gases toward a higher $F_{O_{2}}$/$F_{N_{2}}$ ratio, driven by hyperventilation. An uneven distribution of error in the transient phase is known to have a disproportionate influence on measurement of gas exchange kinetics (Whipp & Rossiter, 2005), bringing into question whether the IND algorithm provides the solution for improving accuracy of kinetic analyses.

## 2 MASS BALANCE IN ALVEOLAR OXYGEN UPTAKE

Although the IND algorithm is currently not widely adopted in clinical or research laboratories, its use raises several concerns. One of these is a discrepancy in mass balance. This issue is evident when combining Equation 1 and Equation 2, which, for a single breath, yields (Cettolo & Francescato, 2018; Francescato & Cettolo, 2019):

(3) $$\begin{array}{ccc} V_{O_{2i}A} & = & \int_{t1_{i}}^{t2_{i}}\left({\dot{V}}_{I}-{\dot{V}}_{E}\right)\cdot F_{O_{2}}\cdot dt-\frac{F_{O_{2}\left(t1_{i}\right)}}{F_{N_{2}\left(t1_{i}\right)}}\cdot \int_{t1_{i}}^{t2_{i}}\left({\dot{V}}_{I}-{\dot{V}}_{E}\right)\cdot F_{N_{2}}\cdot \\ dt & = & V_{O_{2i}M}-\Delta V_{O_{2i}S} \end{array}$$

Equation 3 is valid only when the start and end of the IND breathing cycle (i.e., *t*_1_ *_i_* and *t*_2_ *_i_*) coincide with the start and end of a conventional breathing cycle (i.e., the start of two consecutive inspirations). However, this coincidence occurs on a limited number of breaths, and falls even further when ventilation is rapidly changing. Thus, in order that mass balance of O_2_ flow from the atmosphere to the tissues be preserved, the characteristic overlaps and gaps between breaths with the IND algorithm necessitate the application of an O_2_ volume correction factor (*C_i_*):

(4) $$\begin{array}{ccc} V_{O_{2i}A} & = & \int_{t1_{i}}^{t2_{i}}\left({\dot{V}}_{I}-{\dot{V}}_{E}\right)\cdot F_{O_{2}}\cdot dt-\frac{F_{O_{2}\left(t1_{i}\right)}}{F_{N_{2}\left(t1_{i}\right)}}\cdot \int_{t1_{i}}^{t2_{i}}\left({\dot{V}}_{I}-{\dot{V}}_{E}\right)\cdot F_{N_{2}}\cdot \\ dt\pm C_{i} & = & V_{O_{2i}M}-\Delta V_{O_{2i}S} \end{array}$$

*C_i_* results from the subtraction or summation of the O_2_ volume pertaining to an overlap or gap between the breath *i* and *i +* 1 (Figure 2b and 2c), respectively:

(5) $$C_{i}=\int_{j1_{i}}^{j2_{i}}\;{\dot{V}}_{E}\cdot F_{O_{2}}\cdot dt\;-\frac{F_{O_{2}\left(t1_{i}\right)}}{F_{N_{2}\left(t1_{i}\right)}}\;\cdot \;\int_{j1_{i}}^{j2_{i}}{\dot{V}}_{E}\cdot F_{N_{2}}\cdot dt$$

where ${\dot{V}}_{E}$ and $F_{O_{2}}$ refer to the instantaneous absolute expired gas flow and fractional gas concentrations of O_2_, respectively. In Equation 5, the integration time between *j*_1_ and *j*_2_ represents the duration of the overlap or gap portion between two consecutive IND breaths. It is important to note that the O_2_ volume resulting from the integration of gas flow and O_2_ concentration for overlapped breaths must have a negative sign, as these values need to be subtracted from the computation to correct Equation 4. The opposite occurs for the volume resulting from the integration of gas flow and O_2_ concentration for gaps between IND breaths, as these values need to be summed to Equation 4 to account for the missed O_2_ volumes. Of note, Equation 5 relies on the assumption that overlaps and gaps occur exclusively during the expiratory phase. An example of the O_2_ volumes computed over a series of gaps and overlaps is presented in Figure 3a.

A similar volume correction approach was previously implemented in Grønlund's algorithm (GrØnlund, 1984), which, like the IND algorithm, solved for alveolar B‐by‐B gas exchange based on a redefinition of the breathing cycle. However, in that case, the proposed correction relied on assumptions that gas exchange within the correction period is negligible and that the individual breath respiratory exchange ratio (RER = ${\dot{V}}_{CO_{2}}$/${\dot{V}}_{O_{2}}$; i.e., carbon dioxide output divided by oxygen uptake) equalled 1—assumptions that are rarely fulfilled (Capelli et al., 2011; Girardi et al., 2023).

It is not disputed that one benefit of the IND algorithm is to reduce B‐by‐B variability in $V_{O_{2}}$ (Francescato & Cettolo, 2019; Francescato et al., 2019), because IND ${\dot{V}}_{O_{2}}$ more faithfully tracks $V_{O_{2i}A}$ than does $V_{O_{2i}M}$. However, it achieves this by introducing a known, non‐systematic error that does not conserve O_2_ mass balance on a B‐by‐B basis. These effects are particularly pertinent during exercise transition phases where gas concentration profiles change rapidly (Allen & Jones, 1984; Steinacker et al., 2001). By ignoring the gas volume correction, *C_i_*, the IND algorithm fails to conserve mass balance of O_2_ (or CO_2_) at different physiological locations of measurement. Because tissue respiration drives the demand of O_2_ exchange, and tissue respiration does not occur on a B‐by‐B basis, this correction factor cannot be ignored. Current standard B‐by‐B gas exchange measurements forfeit measurement of alveolar exchange for $V_{O_{2i}M}$ in the knowledge that ${V_{O_{2}}}_{M}$ contains an unknown (Δ $V_{O_{2i}S}$), but that it maintains accuracy and conserves mass balance. These concerns raise significant questions about the conceptual validity of the IND algorithm to appropriately quantify O_2_ flow across the alveolar compartment in relation to O_2_ transport from the atmosphere to the tissues.

## 3 INTERPRETING BREATH‐BY‐BREATH GAS EXCHANGE IN A WORLD OF DISCONTINUOUS BREATHING CYCLES

Our proposed correction, *C_i_*, accurately overcomes errors in $V_{O_{2i}A}$ and $V_{CO_{2i}A}$ associated with gaps and overlaps to preserve mass balance using the IND algorithm. Nevertheless, this mitigates only one challenge inherent with the use of these algorithms. Additional challenges arise with accepting an alternative definition of the breathing cycle on other exercise‐related constructs and on clinical interpretation of CPET results. The authors of the IND algorithm have stated that their solution for $V_{O_{2i}A}$ and $V_{CO_{2i}A}$ was designed to reduce variability in B‐by‐B gas exchange measurements by accounting for changes in lung gas stores, but was not designed to be applied to the B‐by‐B calculation of other key breathing‐related variables (Francescato & Cettolo, 2019, 2024, 2024b). Nevertheless, there exist several additional challenges of accepting an alternative definition of the breathing cycle that highlight the logical limitations of accurately estimating lung gas stores using the IND algorithm.

Unlike conventional methods that measure B‐by‐B ${V_{O_{2}}}_{M}$ and ${V_{CO_{2}}}_{M}$ within the same breathing cycle, the IND algorithm assesses volumes of O_2_ and CO_2_ exchange over different, independent time frames and flow rates, which bear no relation to the physiological breathing cycle. This originates from calculating gas volumes through two separate traces: $V_{O_{2i}A}$ from the $F_{O_{2}}$/$F_{N_{2}}$ ratio and $V_{CO_{2i}A}$ from the $F_{CO_{2}}$/$F_{N_{2}}$ ratio (Girardi et al., 2023). Thus, the ‘breath’ that captures $V_{O_{2i}A}$ data does not temporally align with the ‘breath’ used for $V_{CO_{2i}A}$ measurements. Linking these two disparate gas volumes introduces error into the calculation of RER.

RER is integral to a range of applications in research and clinical practice (American Thoracic Society & American College of Chest Physicians, 2003; Sietsema & Rossiter, 2023; Sietsema et al., 2021). These include the estimation of tissue carbohydrate and lipid oxidation rates at rest and in exercise. In addition, RER is a variable used in calculation of the alveolar–arterial $P_{O_{2}}$ difference ($P_{\left(A-a\right)O_{2}}$):

(6) $$\begin{array}{ccc} P_{AO_{2}} & - & P_{aO_{2}}=\left[F_{IO_{2}}\times \left(P_{B}\;-47\right)-\frac{P_{\mathrm{AC}O_{2}}}{\mathrm{RER}}+\frac{P_{\mathrm{AC}O_{2}}\times F_{IO_{2}}\times \left(1-\mathrm{RER}\right)}{\mathrm{RER}}\right]\\ & & -\;P_{aO_{2}} \end{array}$$

where $F_{IO_{2}}$ is the fraction of inspired O_2_, the term $\left(P_{B}\;-47\right)$ is the barometric pressure minus a constant accounting for partial pressure of water vapour at body temperature, and $P_{\mathrm{AC}O_{2}}$ is the alveolar partial pressure of CO_2_. (Note that Equation 6 assumes a negligible quantity of CO_2_ in the inspired gas; when CO_2_ is present in the inspired air, a different equation should be used; Rahn & Fenn, 1955). $P_{\left(A-a\right)O_{2}}$ is the gold‐standard index of pulmonary O_2_ exchange efficiency, widely used at rest and during exercise in both athlete and patient populations, and also in the intensive care unit to prescribe inspired oxygen requirements for management of arterial hypoxaemia. Importantly, however, Equation 6 cannot be solved when using alternative definitions of the breathing cycle because RER is not appropriately resolved.

A further concern is that redefining the breathing cycle poses substantial challenges for accurately defining timing‐ and ventilatory‐based variables that are fundamental in research, clinical settings and CPET interpretation, including pulmonary ventilation (${\dot{V}}_{E}$), ventilatory equivalents for O_2_ and CO_2_ (${\dot{V}}_{E}$/${\dot{V}}_{O_{2}}$ and ${\dot{V}}_{E}$/${\dot{V}}_{CO_{2}}$), tidal volume (*V*_T_), breathing rate (BR), and inspiratory and expiratory times (Girardi et al., 2023; Rossiter & Poole, 2023; Ward, 2018; Whipp et al., 2005). Compounding this complexity is the fact that some of these variables have two solutions using the IND algorithm—one from breathing cycles identified using the $F_{O_{2}}$/$F_{N_{2}}$ ratio and another from those identified with the $F_{CO_{2}}$/$F_{N_{2}}$ ratio. This incongruity raises a key dilemma of which set of the duplicated measurements (if any)—for example, ${\dot{V}}_{E}$, *V*_T_ or BR—should be used in clinical applications. In CPET, primary and derived variables are assessed in combination to identify clinical abnormalities, for example, in oxygen delivery and utilization, mechanics of breathing, cardiac function, and pulmonary gas exchange efficiency. Wasserman's nine‐panel plot (Sietsema et al., 2021) is an invaluable example of the integrative interpretative power of CPET response profiles, which is lost when applying alternative non‐physiological definitions of the breathing cycle. This would complicate clinical decision‐making processes, diagnostic and prognostic stratification, and measurement of treatment efficacy.

Knowledge of the dead space fraction of a tidal breath (*V*_D_/*V*_T_) is essential for understanding pulmonary gas exchange efficiency at rest and during exercise, especially in patient populations (American Thoracic Society & American College of Chest Physicians, 2003; Sietsema et al., 2021). *V*_D_/*V*_T_ can be estimated using measurements of arterial and mixed expired CO_2_ partial pressures (i.e., the Enghoff modification of the Bohr equation), or using B‐by‐B measurement of *V*_T_, ${\dot{V}}_{E}$/${\dot{V}}_{CO_{2}}$ and transcutaneous partial pressure of CO_2_ ($P_{\mathrm{tcC}O_{2}}$) (Cao et al., 2021; Porszasz et al., 2018; Weatherald et al., 2018). It remains uncertain whether these calculations are reliable when using alternative breathing cycle definitions.

Perhaps most significantly, the investigation of the physiological mechanisms that control breathing and gas exchange requires a physiologically meaningful definition of the breathing cycle. The time at which expiratory gas flow is inhibited and inspiratory gas flow begins is a neural–mechanical coupling event uninfluenced by $F_{O_{2}}$/$F_{N_{2}}$ or $F_{CO_{2}}$/$F_{N_{2}}$ ratios (Del Negro et al., 2018). Investigation of the neural control of breathing cannot reasonably cohere with such alternative breathing cycle definitions, acceptance of which would be at odds with established neurophysiological principles.

## 4 FUTURE DIRECTIONS

Several studies show that algorithms accounting for changes in lung gas stores reduce B‐by‐B variability in gas exchange measurements (Capelli et al., 2011; Girardi et al., 2023). Data also suggest that ignoring lung gas store changes in B‐by‐B analysis introduces distortions in gas exchange kinetics (Capelli et al., 2011; Cautero et al., 2002). For example, the ${\dot{V}}_{O_{2}}$ mean response time is faster when measured as alveolar ${\dot{V}}_{O_{2}}$ using the IND algorithm compared to that from standard methods (Francescato & Cettolo, 2024a). Similar findings have been reported with other algorithms that account for changes in lung gas stores (Capelli et al., 2011; Cautero et al., 2002). However, the actual alveolar ${\dot{V}}_{O_{2}}$ kinetics are unknown, so there is no gold standard or reference value to establish whether the calculated alveolar ${\dot{V}}_{O_{2}}$ kinetics using IND are indeed more accurate than alternative algorithms.

Computer simulations are an appealing solution to address this issue (Busso & Robbins, 1997). Comparing simulated intra‐breath traces of $F_{O_{2}}$, $F_{CO_{2}}$ and gas flow with different known underlying kinetics across B‐by‐B algorithms could provide a route to establish accuracy. Additionally, comparing these algorithms in awake human subjects under various conditions where lung gas stores, lung volumes, and ventilation–perfusion relationships are expected to change would be of great interest. Although solving the lung gas stores ‘problem’ represents an area in need of further study, the fundamental complexity introduced into the interpretation of cardiopulmonary exercise testing variables by accepting an alternative definition of the breathing cycle (e.g., as in IND) speaks against further pursuit of this approach. Focusing on alveolar algorithms based on the conventional definition of the breathing cycle seems more appropriate, as mass balance is preserved without compromising the available variables required for CPET interpretation.

## 5 CONCLUSION

Here, we provide a correction factor that is required to conserve mass balance of gas exchange in algorithms that use an alternative definition of the breathing cycle, such as the IND algorithm (Cettolo & Francescato, 2018; Francescato & Cettolo, 2019). This correction is crucial for accurate measurement of alveolar gas exchange kinetics, which was a key benefit anticipated from the IND approach. Beyond this, however, the IND algorithm is frequently at odds with the traditional definition of a ‘breath’ based on the time between two consecutive inspirations. The consequences of accepting an alternative definition of a breath for measurement and interpretation of the respiratory exchange ratio, pulmonary gas exchange efficiency, dead space fraction of the breath, control of breathing, and a broad spectrum of clinical CPET variables is an impediment to advancing understanding of exercise physiology. For these reasons, we do not support the widespread adoption of alternative definitions of the breathing cycle as a practical solution for alveolar gas exchange measurement in research or clinical settings.

## AUTHOR CONTRIBUTIONS

Michele Girardi and Harry B. Rossiter wrote the first draft of the manuscript. All the authors revisited the work critically for important intellectual content. All authors approved the final version of the manuscript and agreed to be accountable for all aspects of the work in ensuring that questions related to the accuracy or integrity of any part of the work are appropriately investigated and resolved. All persons designated as authors qualify for authorship, and all those who qualify for authorship are listed.

## CONFLICT OF INTEREST

C.F. is involved in contracted clinical research with United Therapeutics, Genentech, Regeneron, Respira Therapeutics and Mezzion. She reports consulting fees from Respira Therapeutics. H.B.R. reports consulting fees from the NIH RECOVER‐ENERGIZE working group (1OT2HL156812), and is involved in contracted clinical research with Astellas, GlaxoSmithKline, Genentech, Intervene Immune, Mezzion, Novartis, Regeneron, Respira, and United Therapeutics. H.B.R. and S.A.W. receive author royalties from sales of *Wasserman and Whipp's Principles of Exercise Testing and Interpretation*, Lippincott, Williams and Wilkins, 6th edn (2020). H.B.R. and C.F. hold visiting academic appointments at the University of Leeds, UK.
